# Population Coding of Forelimb Joint Kinematics by Peripheral Afferents in Monkeys

**DOI:** 10.1371/journal.pone.0047749

**Published:** 2012-10-24

**Authors:** Tatsuya Umeda, Kazuhiko Seki, Masa-aki Sato, Yukio Nishimura, Mitsuo Kawato, Tadashi Isa

**Affiliations:** 1 Department of Developmental Physiology, National Institute for Physiological Sciences (NIPS), National Institutes of Natural Sciences (NINS), Okazaki, Japan; 2 Department of Neurophysiology, National Institute of Neuroscience, National Center of Neurology and Psychiatry, Kodaira, Japan; 3 PRESTO, Japan Science and Technology Agency (JST), Kawaguchi, Japan; 4 Neural Information Analysis Laboratories, Advanced Telecommunications Research Institute International (ATR), Kyoto, Japan; 5 Computational Neuroscience Laboratories, Advanced Telecommunications Research Institute International (ATR), Kyoto, Japan; 6 School of Life Science, the Graduate University for Advanced Studies (SOKENDAI), Hayama, Japan; The University of Western Ontario, Canada

## Abstract

Various peripheral receptors provide information concerning position and movement to the central nervous system to achieve complex and dexterous movements of forelimbs in primates. The response properties of single afferent receptors to movements at a single joint have been examined in detail, but the population coding of peripheral afferents remains poorly defined. In this study, we obtained multichannel recordings from dorsal root ganglion (DRG) neurons in cervical segments of monkeys. We applied the sparse linear regression (SLiR) algorithm to the recordings, which selects useful input signals to reconstruct movement kinematics. Multichannel recordings of peripheral afferents were performed by inserting multi-electrode arrays into the DRGs of lower cervical segments in two anesthetized monkeys. A total of 112 and 92 units were responsive to the passive joint movements or the skin stimulation with a painting brush in Monkey 1 and Monkey 2, respectively. Using the SLiR algorithm, we reconstructed the temporal changes of joint angle, angular velocity, and acceleration at the elbow, wrist, and finger joints from temporal firing patterns of the DRG neurons. By automatically selecting a subset of recorded units, the SLiR achieved superior generalization performance compared with a regularized linear regression algorithm. The SLiR selected not only putative muscle units that were responsive to only the passive movements, but also a number of putative cutaneous units responsive to the skin stimulation. These results suggested that an ensemble of peripheral primary afferents that contains both putative muscle and cutaneous units encode forelimb joint kinematics of non-human primates.

## Introduction

Peripheral inputs contribute to kinesthesia, the sense of joint position and movements, and blocking peripheral primary afferents impairs the perception of limb position and movements [1], [2], [3], [4]. More directly, artificial activation of muscle tendons or surface cutaneous receptors induces a powerful illusion of movement [5], [6], [7], [8], [9]. Human studies have shown that peripheral deafferented patients showed error in hand movements without visual feedback [10], [11]. In monkeys that have had the dorsal root transected at the level of the cervical spinal cord, precision grip is severely impaired [12]. Thus, positional information arising from inputs of peripheral afferents is critical to achieve accurate and dexterous movements of the hands and arms of primates.

Microneurographical recordings from humans or single fiber recordings from animals have been conducted to examine the responses of peripheral afferents to mechanical stimuli [13], [14], [15], [16]. Individual sensory receptors were found to respond to movements of the hand or arm [17], [18]. However, recording from a single afferent neuron has limitations for examining the sensory processing from an ensemble of peripheral receptors during the dynamic movements. To understand the neural processing of natural movements, it is requisite to simultaneously record the activity of a population of peripheral afferents and to investigate the computation required for those multiple receptors to represent the kinesthesia.

Recent advances in multichannel recordings allow the simultaneous detection of the activity of neuronal ensembles at nerve bundles or dorsal root ganglions (DRGs) [19], [20], [21]. Analysis of the activity of peripheral afferent populations in cat lumbar DRGs allowed the reconstruction of the kinematic state of the leg accurately using the linear regression model [22], [23], [24]. The results of these studies demonstrate that populations of sensory receptors contain rich information that represents various kinematics of each joint in the hindlimb during movements in two dimensional space. However, it remains unclear whether linear models can be used to encode complex and dexterous movements in primates from DRG recordings.

To elucidate the encoding of forelimb position and movements by a population of DRG neurons, we performed multichannel recordings from the DRGs in the lower cervical segments of anesthetized monkeys. We applied the sparse linear regression (SLiR) algorithm to encode the forelimb joint kinematics from activities of DRG neuronal ensembles. The SLiR effectively and automatically selected appropriate feature sets from thousands of parameters to attain an improved generalization of performance over that obtained from other ordinary linear regression models [25], [26]. By selecting the optimal ensemble from recorded units, the analysis may reveal the underlying physiology in encoding of joint kinematics. We classified the recorded units into two groups, putative muscle and cutaneous units, by the response property to peripheral mechanical stimulations. We then analyzed neuronal populations selected by the SLiR, and examined the contribution of the two groups to the encoding of joint kinematics.

## Materials and Methods

Two adult male monkeys (Macaca mulatta, *body weight; 4.6 and 9.6 kg, respectively*) were used in this study. The experiments were approved by the animal experimental committee of the National Institute of Natural Sciences (Approved Nos.: 09A196, 10A203, 11A168) and were performed in accordance with the Weatherall report, “The use of non-human primates in research”. Before the experiments, the animals were housed individually on a 12-hour light/dark cycle and provided a rubber toy. They were not food and water deprived.

### Preparation

The monkeys were sedated using a mixture of xylazine (0.4 mg/kg; Bayer Health Care, Monheim, Germany) and ketamine (5 mg/kg; Daiichi Sankyo, Tokyo, Japan), and then anesthetized with isoflurane (exhaled level; 1–2%) and nitrous oxide gas (1–2%). Atropine (0.1 mg/kg; Mitsubishi Tanabe Pharma, Osaka, Japan) and dexamethasone (0.15–0.3 mg/kg; Banyu, Tokyo, Japan) were administered intramuscularly immediately after the anesthesia. The animals were paralyzed using pancuronium bromide (Mioblock; 0.2 mg/kg/h; Schering-Plough Corporation, Kenilworth, NJ), and artificial pneumothorax was introduced and artificial respiration was provided. Expiratory CO_2_ levels were monitored continuously and maintained within the physiological range (3.3–4.2%). Blood pressure was maintained above 80 mmHg. The depth of anesthesia was monitored continuously by checking the stability of blood pressure, heart rate and lack of pupillary reflex.

**Figure 1:**
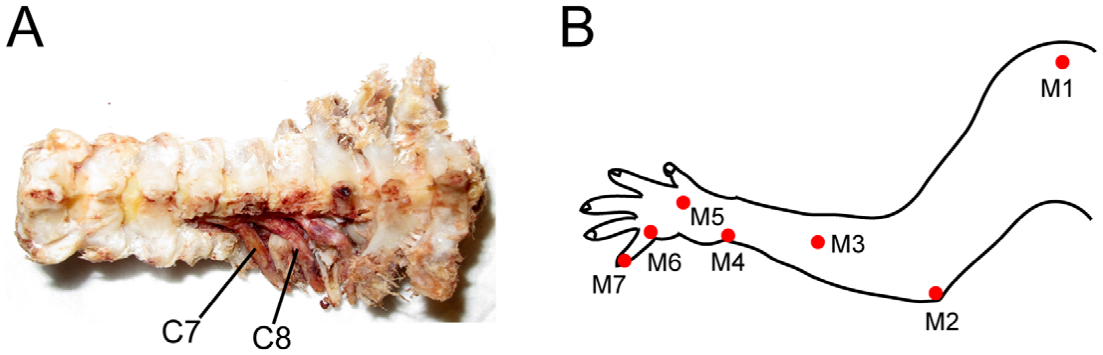
Experimental setup. ***A***, Dorsal view of the spine and dorsal root ganglions (DRGs) in the cervical segments of a monkey. The cervical (C2) through thoracic (Th3) vertebrae are shown. Microelectrode arrays were implanted in the left C7 and C8 DRGs. ***B*** **,** Marker placements on the left arm and hand.

After shaving the hair on the back and the left forelimb, a partial laminectomy was performed to expose the DRGs at the C7 and C8 segments. A lateral mass of C5–Th1 segments was dissected. Two multi-electrode arrays (Blackrock Microsystems, Salt Lake City, UT) were inserted with a high-velocity inserter [27] through the dura into the C7–C8 DRGs on the left side (Fig. 1A). Reference wires were placed into the back muscles. After surgery, the monkeys were suspended in a spinal frame and radiant heat was used to maintain body temperature near 37°C. After finishing the recording session, the animals were deeply anesthetized using pentobarbital sodium (100 mg/kg, intravenous injection) and perfused transcardially with 0.1 M phosphate buffered saline and 10% formalin (Nacalai tesque, Kyoto, Japan), and the placement of the electrode arrays into the DRGs was confirmed.

### Neural Recording and Spike Detection

The implanted arrays consisted of 48 platinized**-**tip silicon electrodes (100–1,000 kΩ at 1 kHz), arranged in a square grid (400 µm on center), 1 mm in length, and in a 5×10- configuration [28]. The size of the array covered a DRG of 2–3 mm in diameter and 4 mm in length. The electrode arrays were connected to a 128-channel amplifier (Cerebus; Blackrock Microsystems) with a gain of 1000, and signals from each electrode were sampled at 30 kHz. Filtered waves (250–7500 Hz) above the amplitude threshold, which is 5 times the estimated background noise based on the median of the absolute value of the bandpass filtered signals [29], were extracted from 0.33 ms before to 0.73 ms after threshold crossing. Spikes with similar features on the principal component analysis (PCA) projection were grouped into clusters by semi-automatic spike sorting methods (Offline sorter; Plexon, Dallas, TX). If an interval between two consecutive units was less than 1 ms, we used first spikes for analysis of unit activity, even though these constituted a small population. Although a portion of the units (14.3% in Monkey 1, 8.7% in Monkey 2) responded to stimulation of distinct areas located far from one another, and were considered to be mixtures of more than one neuron, most of the units were the activity of one neuron.

In some analyses, we excluded the contamination of multi neuronal activity by setting the amplitude threshold as larger values in each channel. Inter-spike intervals of units isolated by the sorting method were more than 1 ms, which implied no contamination from other neurons. Neuronal firing rates for each unit were computed at 5-ms bins, corresponding to the sampling rates of the motion capture system.

### Motion Capture

**Figure 2:**
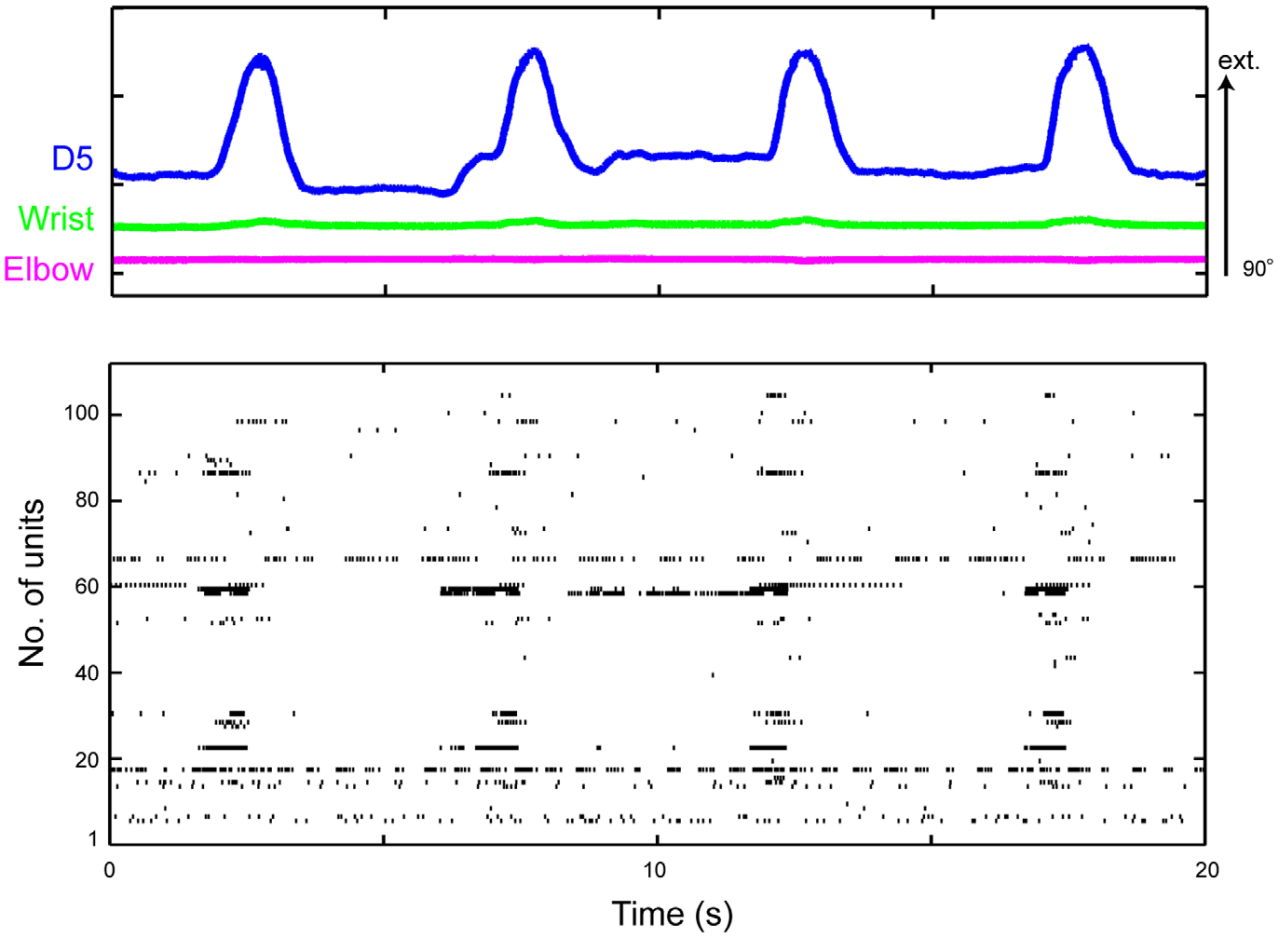
Simultaneous recording of DRG neuronal ensemble activities and elbow/wrist/finger joint kinematics from an anesthetized monkey. *(top)* Elbow, wrist, and digit 5 (D5) MCP joint angles. Extension is represented by an upward deflection (arrow) of the traces shown. Length of the arrow represents magnitude of the angle. *(bottom)* Activities of 112 simultaneously recorded units in the C7 and C8 DRGs during simple extension and flexion movements of the finger joint.

Movements of the upper limb, from shoulder to fingers, were recorded using reflective markers tracked with an optical motion capture system (Eagle-4 Digital RealTime System; Motion Analysis, Santa Rosa, CA) and synchronized with the neural recordings. The system used 9 or 6 infrared cameras operating at 200 frames/s to track the position of multiple reflective markers (6-mm-diameter spheroids) with submillimeter accuracy in Monkey 1 and 2, respectively. A total of 7 markers were attached to the dorsal side of the forelimb using mild adhesive (Fig. 1B), and a comprehensive catalog of 3 anatomically defined upper extremity joint angles (elbow, wrist, finger) were analyzed (Fig. 2). In particular, Euler angles were used to represent relative joint rotations. Elbow joint angles were calculated from two vectors (one from marker 2 to marker 1 and the other from marker 2 to marker 4 in Monkey 1; one from marker 2 to marker 1 and the other from marker 2 to marker 3 in Monkey 2). Wrist joint angles were calculated from two vectors (one from marker 4 to marker 2, a cross product of a vector from marker 4 to marker 6, and the other from marker 4 to marker 5). Finger joint angles were calculated from two vectors (one from marker 6 to marker 7, a cross product of a vector from marker 4 to marker 6, and the other from marker 4 to marker 5). The trajectory of an endpoint of the limb was obtained by subtracting the position of marker 1 from that of marker 7 in the Cartesian coordinate system (from caudal to cranial (CC), from distal to proximal (DP), and from ventral to dorsal (VD)). To reduce noises from various sources, temporal changes in the joint angles and the position of the endpoint of the limb were smoothed using a 5 Hz cutoff frequency in a low-pass digital filter. For convenience, we refer to the first and second time derivatives of joint angles and the position of the endpoint as ‘velocity’ and ‘acceleration’, respectively.

### Mechanical Stimulations

The left forelimb, from forearm to digits, was moved manually. In single joint movements, passive movements were applied to one of the 3 forearm joints: the elbow, wrist, and metacarpophalangeal (MCP) joints of digit 5 (D5). These joints were repeatedly moved from the neutral position (elbow joint angle 90°, wrist 90°, finger 90°) toward the extension or flexion direction and back to the original position. Monkey 2 also underwent pronation. One trial took 5 s in Monkey 1 and 3 s in Monkey 2, from 2 s and 1 s before the stimulation onset, respectively. One session was composed of 26–30 trial repetitions. To investigate more complex, compound movements, the left forearm of Monkey 1 was moved sequentially and arbitrarily by an experimenter at 7 forearm joints, including the elbow, wrist, and MCP joints of the 5 digits for 10 min. Compound movements of the forearm were performed in 2 sessions. Tactile stimulations were applied 29–30 times to the skin surface of the forelimb at various sites with a paint brush.

### Linear Regression

Joint angle, velocity, and acceleration were modeled as a weighted linear combination of neuronal activity using a multidimensional linear regression as follows:
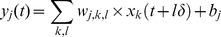
where; y*_j_*(*t*) is a vector of kinematic variables *j* (joint angle, velocity, acceleration of elbow, wrist, finger) at time index *t*. x*_k_*(*t+lδ*) is an input vector of unit *k* at time index *t* and time-lag *lδ* (*δ* = 5 ms). w*_j,k,l_* is a vector of weights on unit *k* at time-lag *lδ*, and b*_j_* is a vector of bias terms to y*_j_*. The units that showed no more than one spike in the training data sets were omitted before the regression analysis. Because external stimulation was the cause of any afferent activity, time-lag was set at future, positive values. According to the goodness of reconstruction of the kinematic data from the neural activity as a function of the input-signal duration, we set the duration of signals at 100 ms (maximum *l* = 20). When we changed the length of the time window, the plateau level in the accuracy of estimation of joint kinematics was achieved at 100 ms. If we consider the conduction velocity of afferent nerves to be more than 10 m/s [30] and their length to be ∼30 cm, 100 ms is presumed to be sufficient. This is probably because good prediction of the encoding of joint kinematics for 3-dimensional movements required sufficient amounts of DRG activity, but the firing frequency of individual DRG neurons was quite low. A set of linear weights were estimated by the linear regression method from a set of training input (unit activity) and output (kinematic variables) data. For comparison with the SLiR method, we also adopted a regularized linear regression method. The regularization parameter was determined from the data using a Bayesian estimation method. After low-pass filtration of the acquired reconstruction at 5 Hz, we compared the predicted kinematics to the observed kinematics for validation of the model.

### Sparse Linear Regression (SLiR)

An SLiR algorithm used in other studies [26], [31], [32], [33], [34], [35], [36] was applied to analyze the population coding of DRG neurons. The SLiR effectively and automatically selected appropriate feature sets and pruned less important signals from thousands of explanatory variables to attain a better generalization performance compared to the regularized linear model. This is because having too many parameters relative to the limited number of training data sets is known to lead to poor generalization performance (over-fitting problem) [37], [38]. We used a Bayesian SLiR algorithm that introduced sparse conditions only for the unit dimension, and not for the temporal dimension (see 35). This method estimated the weight and the automatic relevance determination (ARD) parameters, which represented how much the weight contributes to the reconstruction. Based on the values of the ARD parameters, relevant features were selected automatically and irrelevant features were discarded.

### Data Analysis

In regression analyses of single joint movements, an SLiR model was generated for each of the kinematic parameters investigated (joint angle, velocity, and acceleration). The models generated in the training data sets were tested against a test data set. The training data sets were composed of 40 randomly selected trials (20 extension movement trials and 20 flexion movement trials) for each joint movement (a total of 120 trials). The test data sets were composed of 6 randomly selected trials (3 extension trials and 3 flexion trials) in each joint movement (total of 18 trials). As the detection of some reflective markers were missed in Monkey 2, data of wrist flexion, pronation, and digit 5 joint extension and flexion movements were used in wrist joint encoding, and data of wrist flexion and digit 5 joint extension and flexion movements were used in digit 5 joint encoding. In regression analyses of compound movements, continuously recorded data were partitioned into 20 trials (one trial for 25 s data). Among the 20 trials, 17 randomly selected trials were used for the training data sets, and the remaining 3 trials for the test data sets.

To assess model generalization, we used data from 3 different movement blocks (single joint movements and compound movement sessions 1 and 2) of Monkey 1. The test data sets were built from the different movement blocks of the training data sets. For example, a model was constructed from compound movement session 1, and the model was tested using a data set from session 2.

In characterizations of units selected by the SLiR, we surveyed the training and test data sizes to evaluate the test performance. When the test performance was evaluated using a limited number of data samples, there were trade-offs between the test performance and accuracy of the evaluation. The test performance increased as the training data size increased, while the accuracy of the test performance evaluation increased with increasing test data size. Therefore, we evaluated the balance between the training and the test data sizes by changing the sizes of the training and test data sets. To do this we constructed data sets from combinations of the different movement blocks of Monkey 1, using 150 5-s trials from the single joint movements and 100 5-s partial trials from each session of compound movements to construct pooled data totaling 350 trials.

To quantify the generalization and survey the training and test data sizes, we applied simultaneously the SLiR to data composed of 9 joint kinematics data (angle, velocity and acceleration of each of 3 joints). In other cases, we applied individually the SLiR to the kinematics data of each joint.

**Figure 3:**
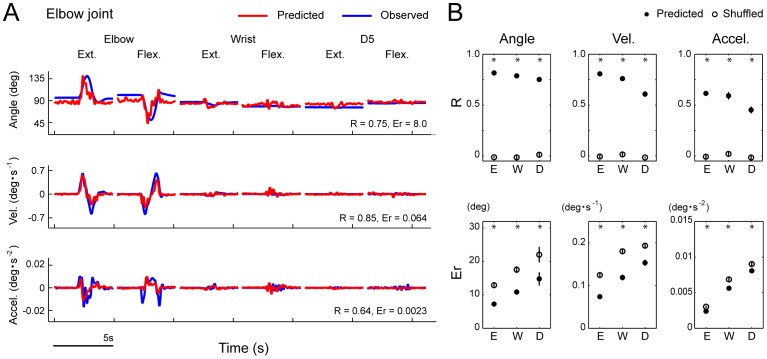
Performance of the SLiR in predicting joint kinematics from DRG activity in single joint movements. ***A*** *,* Observed kinematics of the elbow joint (blue) and their prediction using the SLiR model (red) during 6 different single joint movements in Monkey 1: elbow extension and flexion (Elbow ext.), elbow flexion and extension (Elbow flex.), wrist extension and flexion (Wrist ext.), wrist flexion and extension (Wrist flex.), D5 extension and flexion (D5 ext.), D5 flexion and extension (D5 flex.). Shown from top to bottom are the angular changes at the elbow, and its time derivatives (‘Vel.’ and ‘Accel.’). The correlation coefficient (R) and the RMSE (Er) between the observed kinematics and the predicted ones are shown in the lower right corner of each graph. The scale bar shown in the lower left of figures represents 5 s, which is identical to the intervals of two neighboring ticks on the horizontal axis. ***B*** *,* Test performance (correlation coefficient (R) and RMSE (Er)) of the SLiR model in predicting various kinematics of the elbow (E), wrist (W), and D5 MCP joints (D). Indicated values are averages of the results of 10 pairs of training and test data sets from 2 monkeys. To assess the test performance of the recorded data (closed circles), data sets composed of shuffled unit activities were used for the control prediction (open circles). Asterisks indicate that the performance of the SLiR model was significantly different from that of the shuffled data (paired Student’s t-test, p<0.0001). Error bars represent the standard error of the mean (n = 10).

**Figure 4:**
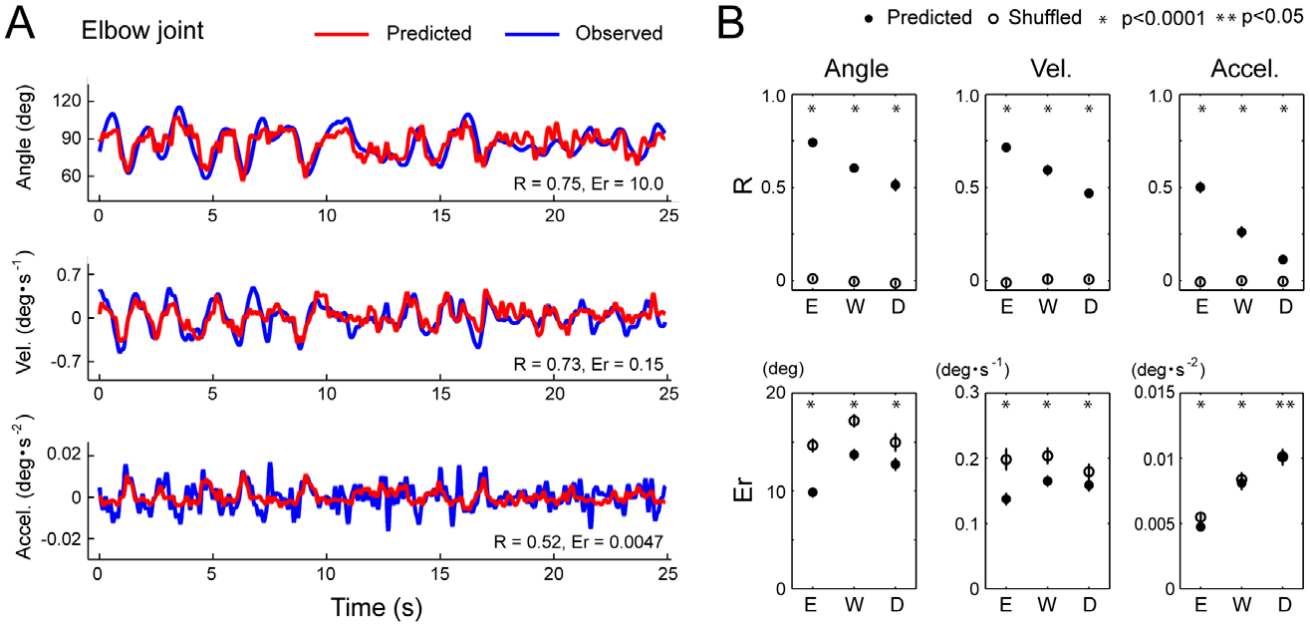
Performance of the SLiR in predicting joint kinematics from DRG activity during compound movements. ***A*** *,* Observed kinematics of the elbow joint (blue) and their prediction using the SLiR model (red) during compound movements. Shown from top to bottom are the angular changes at the elbow, and its time derivatives (‘Vel.’ and ‘Accel.’). The correlation coefficient (R) and the RMSE (Er) between the observed kinematics and the predicted ones are shown in the lower right corner of each graph. ***B*** *,* Test performance (correlation coefficient (R) and RMSE (Er)) of the SLiR model in predicting various kinematics of the elbow (E), wrist (W), and D5 MCP joints (D). Indicated values are averages of the results of 10 pairs of training and test data sets from 2 sessions. To assess the test performance of the recorded data (closed circles), data sets composed of shuffled unit activities were used for control predictions (open circles). Asterisks indicate that the performance of the SLiR model was significantly different from that of the shuffled data (paired Student’s t-test, p<0.0001 or p<0.05). Error bars represent the standard error of the mean (n = 10).

**Figure 5:**
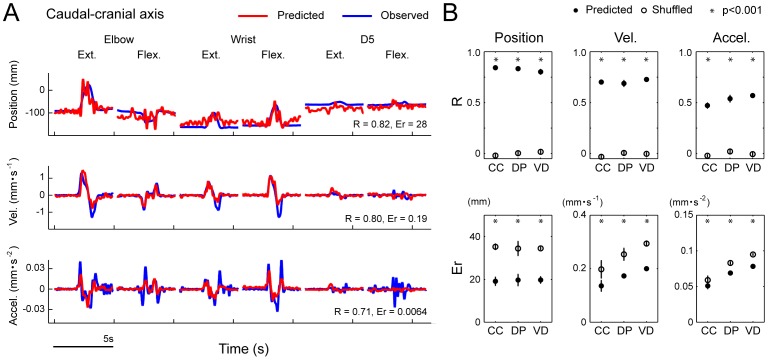
Performance of the SLiR in predicting kinematics of a limb endpoint from DRG activity. ***A*** *,* Observed kinematics of a limb endpoint on the caudal-cranial axis (blue) and their prediction using the SLiR model (red) during single joint movements. Shown from top to bottom are the position, and its time derivatives (‘Vel.’ and ‘Accel.’). The correlation coefficient (R) and the RMSE (Er) between the observed values and the predicted ones are shown in the lower right corner of each graph. ***B*** *,* Test performance (correlation coefficient (R) and RMSE (Er)) of the SLiR model in predicting the endpoint kinematics in the Cartesian coordinate system (caudal-cranial (CC), distal-proximal (DP), ventro-dorsal (VD)). Indicated values are averages of the results of 10 pairs of training and test data sets in single joint movements. To assess the test performance of the recorded data (closed circles), data sets composed of shuffled unit activities were used for control predictions (open circles). Asterisks indicate that the performance of the SLiR model was significantly different from that of the shuffled data (paired Student’s t-test, p<0.0001 or p<0.05). Error bars represent the standard error of the mean (n = 10).

**Figure 6:**
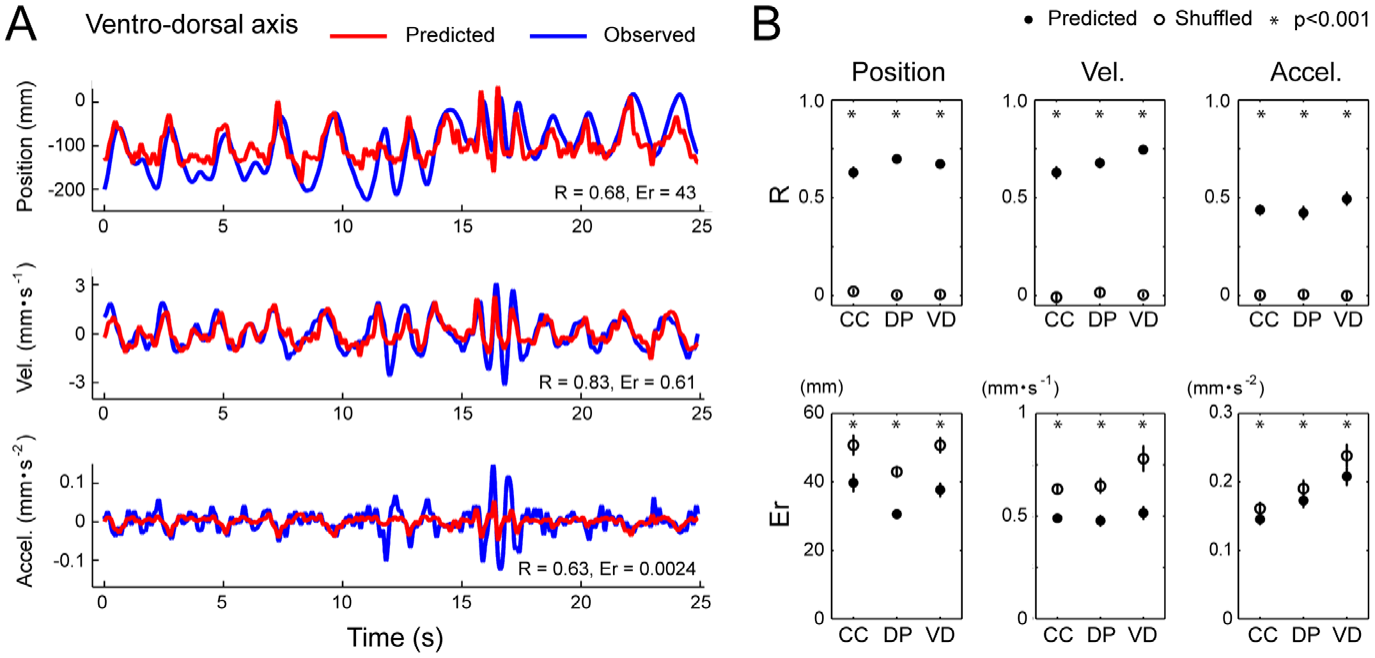
Performance of the SLiR in predicting kinematics of a limb endpoint from DRG activity. ***A*** *,* Observed kinematics of a limb endpoint on the ventro-dorsal axis (blue) and their prediction using the SLiR model (red) during compound movements. Shown from top to bottom are the position, and its time derivatives (‘Vel.’ and ‘Accel.’). The correlation coefficient (R) and the RMSE (Er) between the observed values and the predicted ones are shown in the lower right corner of each graph. ***B*** *,* Test performance (correlation coefficient (R) and RMSE (Er)) of the SLiR model in predicting the endpoint kinematics in the Cartesian coordinate system (caudal-cranial (CC), distal-proximal (DP), ventro-dorsal (VD)). Indicated values are averages of the results of 10 pairs of training and test data sets in compound movements. To assess the test performance of the recorded data (closed circles), data sets composed of shuffled unit activities were used for control predictions (open circles). Asterisks indicate that the performance of the SLiR model was significantly different from that of the shuffled data (paired Student’s t-test, p<0.0001 or p<0.05). Error bars represent the standard error of the mean (n = 10).

Prediction accuracy was evaluated as the correlation coefficient between the observed kinematics of test data sets and the predictions from the model. The root mean squared error (RMSE) between the observed kinematics and the predictions from the model was also calculated to assess the prediction accuracy in some analyses (Figs. 3, 4, 5 and 6). To assess the model, 5 pairs of training and test data sets were generated, and a 5-fold cross-validation was performed in all the analyses. In control analyses of model prediction, we created surrogate training and test data sets in which temporal firing profiles of individual neurons were shuffled independently across different trials and tested subsequently for their prediction of each kinematic parameter.

To infer the importance of individual neurons in reconstruction, we defined two indices: (i) the corresponding weight value determined through the regularized linear regression analysis with population data and normalized by the power of the unit activity in the training data sets, and (ii) the correlation coefficient between the observed kinematics and the predictions derived from single unit activity and corresponding weight values.

In encoding of the joint kinematics by an individual class of sensory neurons, the number of putative muscle units used in the model training was matched to that of the putative cutaneous units to compare the contribution of the putative muscle and cutaneous units. Using random selection, we produced 20 data sets of identical numbers of putative muscle and cutaneous units. We then fit the SLiR using individual data sets and generated a new prediction.

### Statistical Analysis

The data were analyzed using a two-way analysis of variance (ANOVA), the non-directional unpaired- or paired Student’s t-test, with Bonferroni correction if necessary. An alpha level of significance was set at 0.05 for all statistical tests. Data are expressed as the mean ± standard deviation (mean ± S.D.) or the mean ± standard error (mean ± S.E.). We found 95% confidence intervals for proportions based on the inverse of the appropriate cumulative Beta distribution. We used Matlab (Mathworks, Natick, MA) for the statistical analysis.

## Results

Recordings were obtained from the DRGs at the C7 and C8 segments with two multi-electrode arrays. A total of 112 units (39 from C7, 73 from C8) were discriminated from 43 channels in Monkey 1 and 92 units (38 from C7, 54 from C8) were discriminated from 44 channels in Monkey 2.

### Reconstruction of the Forelimb Joint Kinematics from Activities of the DRG Neuronal Ensembles using the SLiR Model

Population recordings during passive movements showed that the temporal discharge patterns of individual isolated units were correlated with temporal changes in the joint angles and that the temporal changes in the firing of each unit varied among the isolated units (Fig. 2).

To examine whether neuronal ensembles in the DRGs convey rich information about joint kinematics, we applied the SLiR model to the encoding of kinematic variables from the activities of all the single and multiple units. As the activation of peripheral afferents was induced by external stimulation, we considered that the peripheral afferents carried information concerning limb position immediately before their firing. Therefore, the kinematic variables were defined as a weighted sum of neural firing for the upcoming 100 ms (here grouped into 20, 5-ms bins) in the SLiR. Figure 3A shows the result of the encoding of elbow joint angle, velocity, and acceleration in a test data set composed of multiple single joint movements, from the activities of a neuronal ensemble. The SLiR provided accurate predictions of the joint kinematics. The prediction performance of the test data sets (test performance) from the actual neural firing pattern was much better than that from the shuffled data (paired student’s t-test; p<0.0001; Fig. 3B).

Next, we applied the SLiR to neuronal activities recorded during compound movements of the forelimb to encode temporal changes of the joint kinematics. Figure 4A illustrates the results of encoding of the elbow joint angles, velocity, and acceleration from ensemble activities in a test data set of compound movements. Even in complicated, three-dimensional movements, prediction of the joint kinematics was accurate. The test performance from the actual neural firing pattern was much better than that from the shuffled data (paired Student’s t-test; elbow and wrist; p<0.0001, finger; p<0.05; Fig. 4B). In the compound movements, a more accurate prediction of the joint kinematics was obtained the greater the distance the joint was from the end of the limb (Fig. 4B). In both the single joint movements and the compound movements, the prediction accuracies of angle and velocity were higher than that of acceleration at the three joints. These results demonstrate that neuronal ensembles in the DRG conveyed rich information about joint kinematics, especially for angle and velocity.

Finally, we tested whether kinematics of the limb endpoint in the Cartesian coordinate system were encoded by the DRG neuronal activity using the SLiR. Examples of encoded kinematics of the endpoint are shown in Figures 5A and 6A in single joint movements and compound movements, respectively. The SLiR also provided accurate predictions of kinematics of the limb endpoint in both movements. The test performance from the actual neural firing pattern was much better than that from the shuffled data (paired student’s t-test; p<0.001; Figs. 5B and 6B). These results demonstrate that the activity of neuronal ensembles in the DRGs also carried rich information about the kinematics of the limb endpoint.

### Improved Generalization in Reconstruction of the Forelimb Joint Kinematics using the SLiR

**Figure 7:**
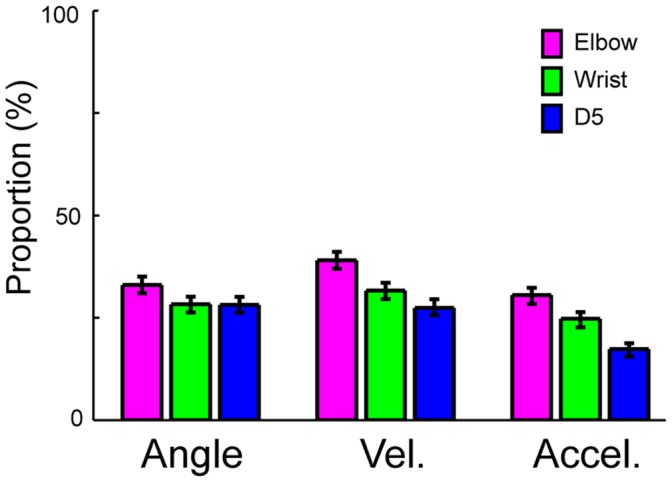
Selection of a subset of DRG neurons by the SLiR. The proportion of units selected by the SLiR in the encoding of joint angle (Angle), velocity (Vel.), and acceleration (Accel.) of the elbow, wrist, and D5 MCP joints. Error bars represent confidence intervals for proportions.

The SLiR reduced the number of inputs used in the prediction (Fig. 7). Note that the numbers of selected units were higher in the prediction of angle and velocity than that of acceleration (paired Student’s t-test with Bonferroni correction (n = 3); p<0.0001), which were correlated with the prediction accuracies of the respective kinematics. In any case, the SLiR accurately encoded temporal changes in the joint kinematics from activities of the DRG neuronal ensembles using reduced numbers of units.

**Figure 8:**
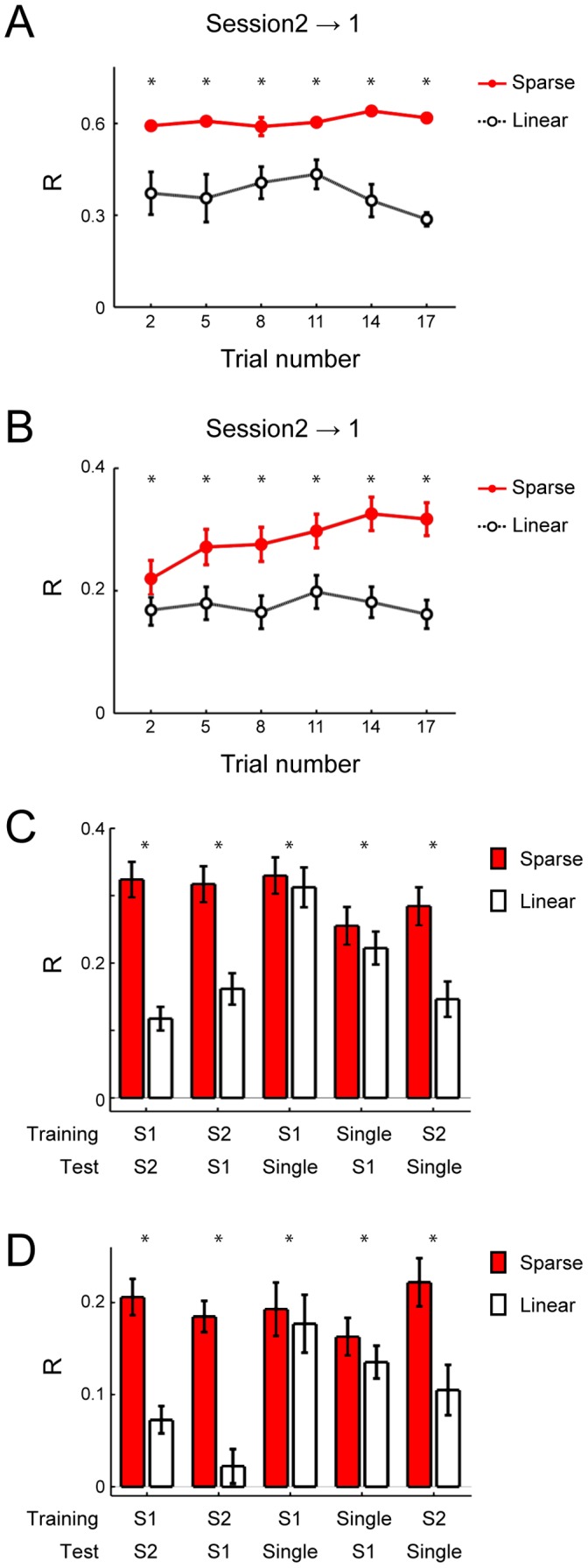
Generalization performances of the SLiR and the regularized linear regression. ***A*** *,* Relationship of the number of training data sets to the correlation coefficient between the prediction and the observation for encoding of the elbow joint angle by the SLiR (red closed circles) and the regularized linear regression (open circles). A decoder was constructed from compound movement session 2, and performance of the decoder was tested using data sets from compound movement session 1. The number of trials in the training data sets was changed as indicated on the horizontal axis. Asterisks indicate significant difference between the performance of the SLiR and the regularized linear regression (paired Student’s t-test, p<0.05). Error bars represent the standard error of the mean (n = 5). ***B*** *,* Relationship of the number of training data sets to the correlation coefficient for encoding of the joint kinematics by the SLiR (red closed circles) and the regularized linear regression (open circles). Indicated values are averages of results in the 9 joint kinematics (angle, velocity, and acceleration of 3 forelimb joints). Asterisks indicate significant difference between the performance of the SLiR and the regularized linear regression (paired Student’s t-test, p<0.05). Error bars represent the standard error of the mean (n = 45). ***C*** *,* Prediction accuracy using the SLiR (red bars) and the regularized linear regression (white bars), when approximately 85% of total trials were used in the training. The movement blocks from which the training and test data sets were built are shown on the horizontal axis. Asterisks indicate significant difference between the performance of the SLiR and the regularized linear regression (paired Student’s t-test, p<0.05). Indicated values are averages of the results in the 9 joint kinematics (angle, velocity, and acceleration of 3 forelimb joints). Error bars represent the standard error of the mean (n = 45). ***D*** *,* The same analysis as shown in ***C*** using units sorted by the more strict sorting rule.

To validate the importance of the units selected by the SLiR, we compared the extent of generalization between the SLiR and the regularized linear regression, which used all of the recorded units to encode the joint kinematics. We used data from 3 different movement blocks (single joint movements and compound movement sessions 1 and 2) of Monkey 1. To assess the generalization of the encoder, an encoder was tested using data sets from the different movement blocks. When an encoder was constructed from compound movement session 2 and the performance of the encoder was tested using data sets from compound movement session 1, the SLiR predicted the joint kinematics more accurately than did the regularized linear regression (Fig. 8A and B). When training data sets were built from approximately 85% of the total data, model performance of the SLiR was statistically better than that of the regularized linear regression model in 5 combinations of training and test data sets (paired Student’s t-test; p<0.05) (Fig. 8C). Thus, the SLiR extracted a limited number of units from the DRG ensemble to encode the joint kinematics with an improved generalization performance.

### Extraction of Appropriate Units to Reconstruct Forelimb Joint Kinematics using the SLiR

Based on a high encoding accuracy with a limited number of units selected by the SLiR, we examined which units were selected from the total sets of recorded neurons. In the following analysis, we used units which were isolated using the more strict sorting rule to reduce the possibility of contamination of any other neuronal activity into isolated single neurons (69 units in Monkey 1 and 74 units in Monkey 2). Using this data set, superior generalization performance of the SLiR was also achieved (Fig. 8D).

**Figure 9:**
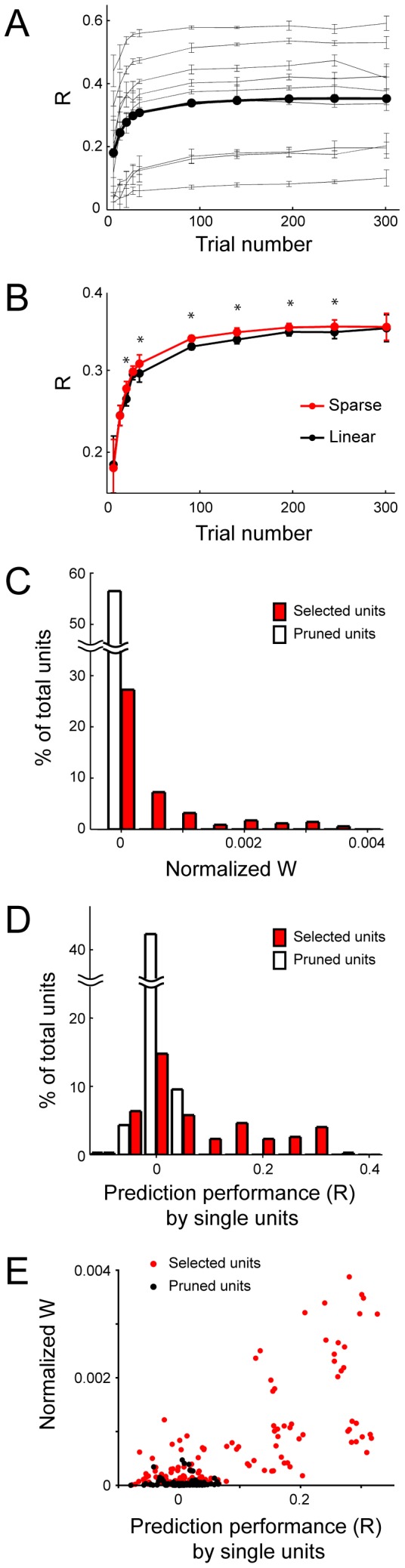
Contribution of units extracted by the SLiR to encoding of the joint kinematics. ***A*** *,* Relationship between the number of trials per training data set and the correlation coefficient between the prediction and the observation for encoding of joint kinematics by the SLiR. Thin gray lines indicate results in encoding of 9 joint kinematics (angle, velocity, and acceleration of 3 forelimb joints), and the thick black line indicates the averages of these results. The number of trials in the training data sets was changed as indicated on the horizontal axis and the remaining trials (the total trial number was 350) were used for the test data sets. Error bars represent the standard deviation. ***B*** *,* Relationship between the number of trials per training data set and the correlation coefficient between the prediction and the observation for encoding of joint kinematics by the SLiR (red circles) and the regularized linear regression (black circles). Each point indicates the averages of results in encoding of 9 joint kinematics. The number of trials in the training data sets was changed as indicated on the horizontal axis and the remaining trials (the total trial number was 350) were used for the test data sets. Error bars represent the standard deviation. ***C*** *,* Distribution of normalized weight values for individual single neurons selected for predicting elbow angle by the SLiR. Histograms are shown for the units selected by the SLiR (red) and the pruned units (white). The total number of analyzed units is 345 (69 x 5 data sets). ***D*** *,* Distribution of the correlation coefficient between the prediction and the observation for encoding of elbow angle from single neurons. Prediction was conducted from the firing rate of each unit and weighted values determined by the regularized linear regression with population data. Histograms are shown for the units selected by the SLiR (red) and the pruned units (white). The total number of analyzed units is 345 (69 x 5 data sets). ***E*** *,* Plots of normalized weight values and correlation coefficient for individual single neurons in encoding of the elbow angle. Each dot represents the result of one single neuron in one block. Plots are shown for the selected units by the SLiR (red) and the pruned units (black). The total number of analyzed units is 345 (69 x 5 data sets).

We first surveyed the training and test data sizes to evaluate the test performance. We analyzed the entire data sets from Monkey 1, because we obtained a larger amount of data sets from this animal than the other. The relationship between the size of the training data sets and the prediction accuracy indicated that the performance achieved a plateau at 140 trials (corresponding to recording for 700 s) per training data set (Fig. 9A and B). At the data size, the superior generalization performance of the SLiR was also confirmed (Fig. 9B).

Using 140 trials as the size of the training data set, we calculated two indices based on univariate statistics, (i) weight value normalized to the power of the unit activity and (ii) the correlation coefficient between the observation and the prediction by individual single neuron activity. Figure 9C and D show the distributions of the respective indices for individual single neurons. In both the normalized weight values and the correlation coefficient, units selected by the SLiR were statistically larger than those of the pruned units (Student’s t-test; p<0.0001). The SLiR selected mainly units that contributed to the reconstruction of joint kinematics or determined the outline of temporal changes of these kinematics. Note that some of the selected units had low scores in the univariate analysis, indicating that they contributed less to the reconstruction individually, but might still play important roles as part of the ensemble.

**Figure 10:**
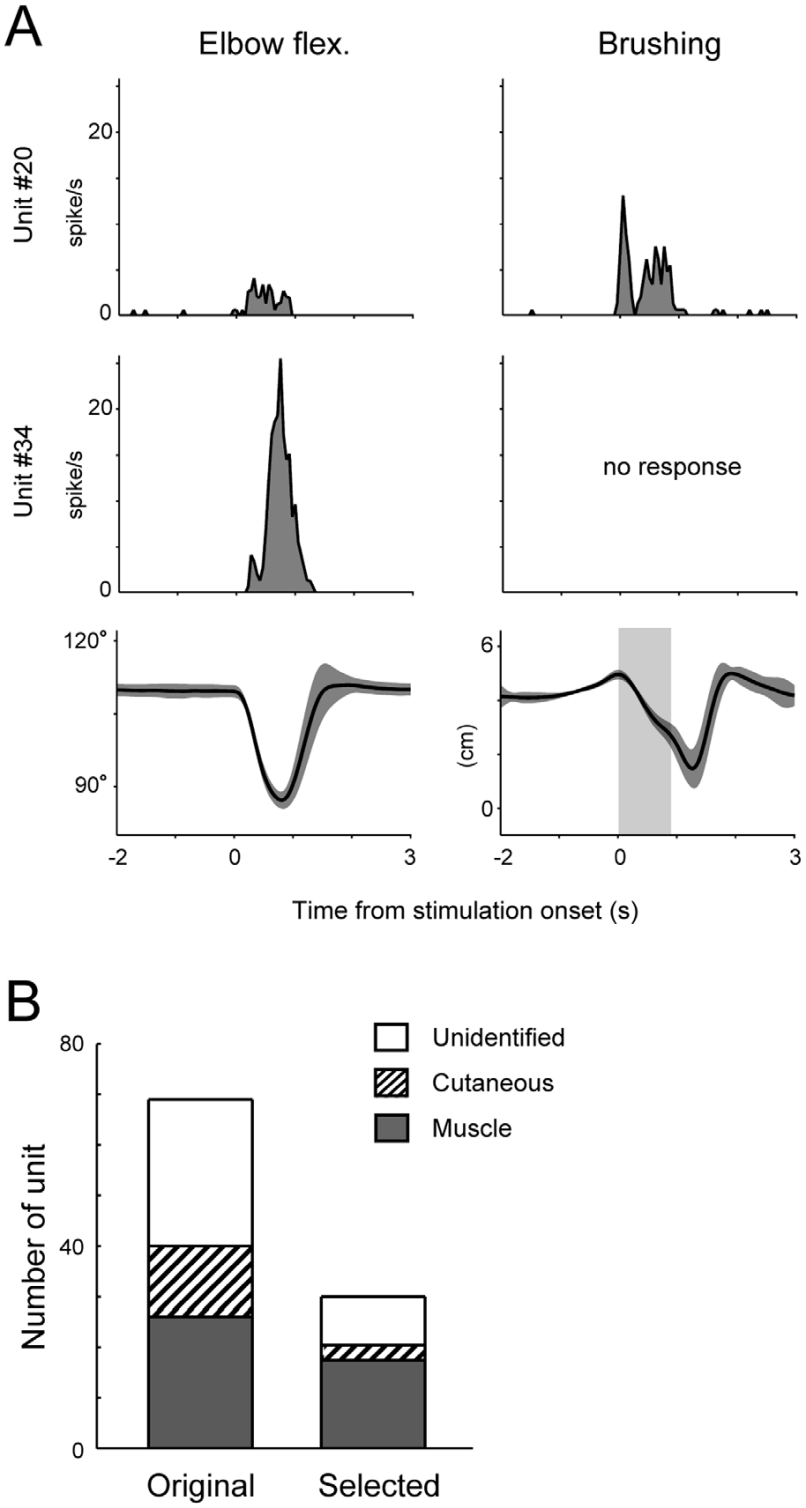
Response property of units selected by the SLiR. ***A*** *,* Peri-stimulus time histograms (bin width 50 ms) for two units recorded during joint movements and skin brushing. Elbow joint angle and positions of the paint brush are shown in the bottom row, in which black lines and dark gray areas represent the mean and standard deviation, respectively. A light gray area represents duration in which the paint brush touched to skin surface. ***B*** *,* Number of all recorded units (original) and those selected units by the SLiR (selected) belonging to the respective class in Monkey 1.

Finally, we examined what DRG neurons were selected by the SLiR. We classified the recorded units into two groups based on their response property to the peripheral stimulations. We first determined whether units responded to the peripheral stimulation by experimenters and then confirmed the presence of statistical significance (p<0.05) between before and during the stimulation using the Wilcoxon signed-rank test. If units responded to a soft brush, they were classified as the putative cutaneous units, while units that responded to passive movements only were classified as the putative muscle units (Fig. 10A). When units did not fire sufficiently for us to categorize them into either of two classes, they were classified as unidentified units. The numbers of units belonging to putative muscle units, putative cutaneous units and unidentified units were 26, 14 and 29, respectively, in Monkey 1, and 41, 22 and 11, respectively, in Monkey 2. The composition of modalities selected by the SLiR for encoding of joint kinematics using 140 training data sets are shown in Figure 10B. Compared to the composition of the originally recorded units, putative muscle units comprised the majority of the selected units (original, 37.7%; selection, 58.0% (50.0 to 65.6 as confidence intervals for proportions)). The SLiR selected a majority of the putative muscle units that were recorded (66.9% (58.4 to 74.4)). These results suggest that putative muscle units contributed much to the encoding of joint kinematics. Although the proportion of putative cutaneous units was reduced following the SLiR selection (21.4% (13.5 to 32.4)), the selected units included a number of putative cutaneous units, suggesting that they also contributed substantially to the encoding of joint kinematics.

### Contribution of the Putative Muscle Units and the Putative Cutaneous Units to Encoding of Joint Kinematics

**Figure 11:**
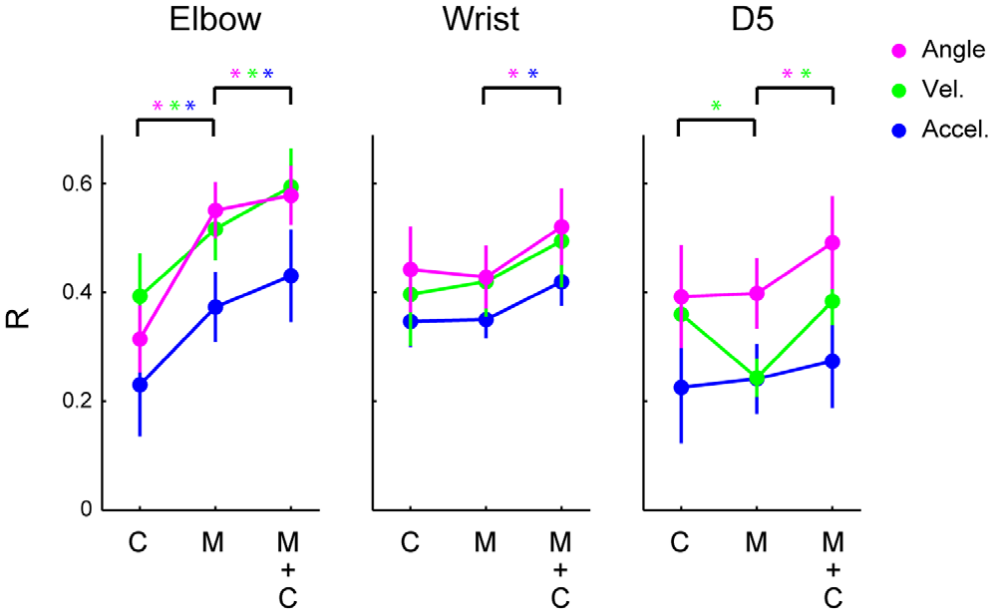
Encoding of joint kinematics from the activities of individual class or combinations of classes. The correlation coefficient between the observation and the prediction by the SLiR using single units of putative cutaneous units only (C), single units of putative muscle units only (M), and their combination (M+C). Indicated values are averages of the results of 10 pairs of training and test data sets from single joint movements of both monkeys and error bars represent the standard error of the mean. Prediction accuracy in the angle is shown in magenta, velocity in green, and acceleration in blue. Asterisks at the top of graphs indicates a significant difference (paired Student’s t-test with Bonferroni correction (n = 3), p<0.05) in the prediction accuracy among neuronal activities of the respective classes.

To confirm that the putative muscle units or the putative cutaneous units individually encode joint kinematics, we applied the SLiR to encoding of joint kinematics from neuronal activities of each category (Fig. 11). Two-way ANOVA revealed significant main effects of category (*F*_(2, 243)_ = 8.2, *p* = 0.0004) and kinematics (*F*_(8, 243)_ = 5.1, *p*<0.00001) with no significant interaction (*F*_(16, 243)_ = 0.76, *p* = 0.73). A post-hoc test showed that the prediction accuracy for encoding of elbow joint kinematics by the putative muscle units activity was higher than that from the putative cutaneous units (paired Student’s t-test with Bonferroni correction (n = 3); p<0.05). To examine the contribution of the putative cutaneous units to encoding of joint kinematics, we compared the prediction accuracy by the SLiR using neuronal activities of both the putative muscle and the putative cutaneous units to that of the putative muscle units alone. By adding the neuronal activity of the putative cutaneous units to those of the putative muscle units, the SLiR selected both the putative muscle and cutaneous units (Tables 1, 2 and 3) and the prediction accuracy in all the kinematics was significantly improved (paired Student’s t-test with Bonferroni correction (n = 3); p<0.05) (Fig. 11). These results suggest that, while a great deal of elbow joint kinematic information was conveyed by the putative muscle units, the putative cutaneous units provided the central nervous system with joint kinematic information that the putative muscle units may not code, such as subtle forelimb movements accompanied with skin deformation.

**Table 1:** Total number of units used in the model and number of units selected by SLiR using single units of putative cutaneous units only.

Joint	Elbow			Wrist			D5		
	Angle	Vel.	Accel.	Angle	Vel.	Accel.	Angle	Vel.	Accel.
Total	13±7.1	13±7.1	13±7.1	13±7.2	13±7.2	13±7.2	13±6.3	13±6.3	13±6.3
Selected cutaneous	8.4±5.8	7.4±3.6	6.3±3.1	5.8±2.8	9.0±3.2	7.5±4.2	6.8±3.6	6.9±2.5	6.3±3.2

The values in the columns are total number of units used in the model (Total) and number of putative cutaneous units selected by the SLiR (Selected cutaneous) in encoding of joint angle (Angle), velocity (Vel.), and acceleration (Accel.) of the elbow, wrist, and D5 MCP joints. Data are expressed as the mean ± standard deviation (n = 10).

**Table 2:** Total number of units used in the model and number of units selected by SLiR using single units of putative muscle units only.

Joint	Elbow			Wrist			D5		
	Angle	Vel.	Accel.	Angle	Vel.	Accel.	Angle	Vel.	Accel.
Total	13±7.1	13±7.1	13±7.1	13±7.2	13±7.2	13±7.2	13±6.3	13±6.3	13±6.3
Selected muscle	8.4±4.2	7.5±3.1	7.4±3.5	6.9±2.8	7.9±4.6	7.8±4.0	6.4±2.5	6.2±3.2	6.0±3.0

The values in the columns are total number of units used in the model (Total) and number of putative muscle unitsselected by the SLiR (Selected muscle) in encoding of joint angle (Angle), velocity (Vel.), and acceleration (Accel.) of the elbow, wrist, and D5 MCP joints. Data are expressed as the mean ± standard deviation (n = 10).

**Table 3:** Total number of units used in the model and number of units selected by SLiR using combination of single units of putative cutaneous units and single units of putative muscle units.

Joint	Elbow			Wrist			D5		
	Angle	Vel.	Accel.	Angle	Vel.	Accel.	Angle	Vel.	Accel.
Total	27±14	27±14	27±14	26±14	26±14	26±14	26±13	26±13	26±13
Selectedcutaneous	7.9±5.2	7.3±3.4	6.4±4.0	6.4±3.4	8.2±2.9	6.8±4.0	6.3±3.3	6.8±2.1	5.0±2.1
Selected muscle	7.6±3.5	7.4±3.1	6.8±2.9	5.7±1.9	6.4±3.1	7.7±3.6	5.5±1.6	4.6±1.7	5.4±2.4

The values in the columns are total number of units used in the model (Total) and number of putative cutaneous (Selected cutaneous) or muscle units (Selected muscle) selected by the SLiR in encoding of joint angle (Angle), velocity (Vel.), and acceleration (Accel.) of the elbow, wrist, and D5 MCP joints. Data are expressed as the mean ± standard deviation (n = 10).

In encoding of wrist and finger joint kinematics, the prediction accuracy from the putative cutaneous units was similar to that from the putative muscle units or superior in encoding of finger velocity (paired Student’s t-test with Bonferroni correction (n = 3); p<0.05). Comparison of the prediction accuracy by the SLiR using neuronal activities of both the putative muscle and the putative cutaneous units to that of the putative muscle units alone also indicated contribution of the putative cutaneous units to encoding of some joint kinematics (paired Student’s t-test with Bonferroni correction (n = 3); p<0.05) (Fig. 11). Furthermore, in the prediction of joint kinematics by the SLiR using neuronal activities of both the putative muscle and the putative cutaneous units, the SLiR selected both of them (Tables 1, 2 and 3). These results suggest that, in wrist and finger joints, the putative cutaneous units provided the central nervous system with joint kinematic information as did the putative muscle units.

## Discussion

The results of the present study demonstrate that temporal changes in angle, velocity, and acceleration of various forelimb joints can be reconstructed from the population activities recorded from cervical DRGs in monkeys using the SLiR. The analysis provided improved generalization performance in the reconstruction of various joint kinematics from a subset of somatosensory neural activity. The analysis elucidated that, not only the putative muscle units, but a number of the putative cutaneous units also contributed to reconstruction of joint kinematics. The result was confirmed by reconstruction of joint kinematics from activity of individual classes of units. The putative muscle units contributed greatly to encoding of elbow joint kinematics, but the putative cutaneous units provided additional information regarding the joint kinematics, especially at wrist and finger joints.

### Multichannel Recording from the Cervical DRGs

The activity of peripheral afferents have been recorded by single fiber recordings in human and animals, in which isolated afferent nerves were recorded by inserting a single microelectrode into the peripheral nerve [13], [14], [15], [16], [17], [39], [40]. These studies identified the response properties of each recorded afferent fiber to various external stimulations or voluntary movements. However, to produce kinesthesia, the sensory information is thought to be assembled in the central nervous system. The coding of positions and movements of the ankle joint by populations of sensory neurons was examined by collecting a number of separate microneurographical recordings during similar movements [41], [42], [43], [44]. Although these studies discriminated movement directions in two dimensional space at a single joint of a leg from the collected recording data, application of the same methodology to the prediction of more complicated natural movements of hand and arm is difficult. For the first time, we recorded simultaneously from approximately 100 sensory neurons using multi-electrode arrays from the cervical DRGs of non-human primates. High variability in the ensemble recording enabled us to calculate various joint kinematics in three dimensional space, especially of compound multi-joint movements. Furthermore, while the microneurographical recording is technically difficult to be performed on subjects who are moving their limbs, we recorded neuronal activity during dynamic movements of the entire forelimb. Thus, the recoding of sensory neural activity using multi-electrode arrays from the DRGs in monkeys affords a new experimental paradigm to explore sensory coding by peripheral afferents as one possible alternative for microneurography.

A series of studies examined the encoding of cat hindlimb movements from ensembles of lumbar DRG neurons [22], [23], [24], calculating position and velocity of the foot in two dimensional space from up to 100 discriminated sensory neurons. We applied a similar recording technique to the DRG neurons at the cervical segments of monkeys, but the forelimb movements in three dimensional space were more complicated than those in the cat studies. Since prediction of movements in three dimensional space from neural activity is more difficult than that in two dimensional space, the prediction accuracy was slightly lower than in the encoding of cat hindlimb movements. On the other hand, prediction of forelimb joint angle or trajectory of the limb endpoint in reaching and grasping movements in three dimensional space from ensemble recordings of the motor cortex provide correlation coefficient values around 0.7 [45], [46], which is similar to the prediction accuracy in our results. Thus, we succeeded in reconstructing elbow, wrist, and finger joints kinematics during movements in three dimensional space using DRG population activity.

### Encoding using SLiR

We demonstrated encoding of forelimb joint kinematics from multichannel recordings of DRG neurons. The elbow and wrist joint kinematics were estimated with higher accuracy than that of the finger. It is possible that higher correlation between the elbow and wrist motions accounts for the higher accuracy in estimation of kinematics of these joints. When the joint movements were correlated, it is difficult to evaluate prediction of kinematics at each joint independently. However, the elbow and wrist motions were not more correlated with each other (0.13 and 0.09 in session 1 and 2, respectively) than other combinations (elbow and finger; 0.33 and 0.32, respectively, wrist and finger; −0.26 and −0.20, respectively). Furthermore, a principal component analysis of joint movement trajectory showed that three joint motions were almost independent in compound movement session 1 (Table 4). In compound movement session 2, their motions were coupled, but the degree of freedom for their motions were three (Table 4). Thus, we conclude that the result of encoding properties of each joint was not dependent on their movement profile, but due to the discharge pattern of recorded ensembles.

**Table 4:** Results of principal component analysis on joint motions in the compound movements.

	Compound movement session 1			Compound movement session 2		
	PC1	PC2	PC3	PC1	PC2	PC3
Elbow	0.13	0.85	−0.50	−0.33	0.64	−0.69
Wrist	0.96	0,02	0,29	0.63	−0.70	0.34
D5	−0.25	0.52	0.82	−0.70	−0.32	0.64

The values in the columns are the eigenvectors of principal component analysis for joint motions. Eigenvalues of three principal components (PC) are (0.49, 0.37, 0.14) in session 1 and (0.44, 0.37, 0.19) in session 2.

The forearm trajectory of the monkey was predicted previously with high accuracy from activities of subsets of motor cortical neurons obtained by multichannel recordings using the SLiR [47]. The authors showed that the SLiR outperformed that of the conventional Wiener filter, which is consistent with our analysis. Their and our present studies suggest that the SLiR selected important units as an ensemble. The ensemble selected by the SLiR was different from units ranked by the conventional univariate analysis, which contained units whose activities were less correlated to joint kinematics as individual units (Fig. 9D). Although some units with weaker correlation showed high contribution to the prediction of joint kinematics (Fig. 9E), these units did not contribute to the whole structure of the joint kinematics but may be relevant to local changes within them. The improved generalization performance of the sparse method has also been accounted for by the correlation structures among ensemble components from the analysis of fMRI data [48].

Reduction of input parameters without any deterioration of coding performance gives a benefit to BMI systems that require less computational burden due to a limited hardware. Arm trajectory can be predicted from the activity of a small number of neurons in the primary motor cortex of primates [49], [50], [51]. The optimal number of neurons to achieve high model performance has been determined by neuron dropping analysis [49], [51]. In this analysis, the linear models were generated from the remaining units after randomly removing a single unit until one neuron was left. The procedure was repeated many times for each ensemble to obtain an optimal ensemble size. One problem is that the method requires significant computational resource and has less practical use in the situation in which composition of chronically recorded units was variable during the experimental period. The SLiR automatically selected important units from the entire recoded population without affecting model performance through machine learning. Thus, the SLiR is a practical method in analyzing data containing an enormous number of recorded neurons.

### Encoding of Joint Kinematics by the Putative Muscle Units and the Putative Cutaneous Units

We classified recorded units into two classes based on their response properties to the peripheral stimulations. Identification of the unit modalities and receptive fields of approximately one hundred units individually as in single afferent recordings is very time consuming. Therefore, we did not apply the identification method to the multichannel recording. Instead, we applied the mechanical stimulation to most of the surface skin of forelimb with a paint brush, and analyzed the response property of each unit after recording. Although the latter offline analysis was not so strict to identify fine receptive field, we considered that the units that responded to tactile stimulation were classified as putative cutaneous units. These units were assumed to be equivalent to cutaneous receptors. The rest of the units that responded to joint movement were classified as putative muscle units, thought to contain muscle spindle, tendon or joint receptors, while muscle spindles may be more commonly encountered. The putative muscle units tended to be more sensitive to passive movements than the putative cutaneous units. Chronic implantation of array electrodes into the DRGs may allow us to identify unit modalities and receptive field definitively.

Muscle receptors are generally considered to be the major source of movement perception. The notion partially stems from the evidence that tendon vibration induces a strong illusion of position and movements [6], [7], [8], [9], [52]. In this study, we showed that the putative muscle units were the major components that encoded the joint kinematics and test performance of elbow joint kinematics prediction was higher from the putative muscle units than the putative cutaneous units. The richness of kinematic information encoded by muscle spindle receptors may be of great value in human psychophysical studies. Nevertheless, cutaneous receptors also encoded additional important positional information. Human experiments showed that specific activation of cutaneous receptors by local stretch and electrical stimulation induced movement illusion [5], [53], [54], [55]. Simultaneous skin stretch and tendon vibration increased the illusory movement compared with vibration only [54]. Thus, the present results that both the putative cutaneous and muscle units contribute to encode joint kinematics are consistent with the results in psychophysical experiments.

Ensembles of cortical neurons in the primary somatosensory cortex in macaque monkeys have provided accurate information about limb kinematics [49], [56], [57]. Cortical neurons in the primary somatosensory cortex are divided mainly into two classes in their receptive fields, cutaneous and proprioceptive receptive fields. Weber and colleagues showed that two classes of cortical neurons contributed equally to encoding of kinematics of the limb endpoint [57]. In the present study, both the putative cutaneous and muscle units also contributed to encoding of joint kinematics at the peripheral level. These results suggest that information about limb kinematics coded by both cutaneous and proprioceptive afferents is conveyed to the cortical neurons with each modality in the primary somatosensory cortex, respectively.

Position sense may be different between distal and proximal regions, especially in the forelimb. The extent of contribution of the putative cutaneous units to encoding of joint kinematics tended to be higher in distal forelimb region than proximal one (Fig. 11). This may be explained by the physical structure of forelimb muscles. As finger muscles, such as the extensor digitorum muscle, control multiple digits, tendon vibration to the finger extensor induces simultaneous illusory movements of these digits [53], [54]. Additional skin stretch to the tendon vibration focuses the sensation of movement to the stretched digit. These results suggested that cutaneous receptors contributed to the positional sense of fingers to a substantial extent. It is well known that two-point discrimination threshold is lower in distal body parts than proximal ones [58]. One explanation of this is that distal parts of the human forelimbs are much more densely innervated by mechanoreceptors than the proximal parts [59]. On the other hand, the number of muscle spindles acting on the joints decreases toward the end of the limbs [60]. Relative high density of peripheral mechanosensory receptors and low density of spindles in the distal forelimb compared to the proximal one may increase the contribution of cutaneous receptor activation to distal kinesthesia compared to those in proximal parts of the body. In addition, results of the present study showed that the putative cutaneous units encoded joint kinematics of distal forelimbs to a greater extent, which suggests that single distal cutaneous units are more important in sensing body position and movements than proximal cutaneous units. These features may constitute high sensitivity to skin deformation in the distal body parts for dexterous manipulation.
